# Prevention of recurrent sickness absence among employees with common mental disorders: design of a cluster-randomised controlled trial with cost-benefit and effectiveness evaluation

**DOI:** 10.1186/1471-2458-10-132

**Published:** 2010-03-15

**Authors:** Iris Arends, Jac JL van der Klink, Ute Bültmann

**Affiliations:** 1 Department of Health Sciences, Work & Health, University Medical Center Groningen, University of Groningen, A. Deusinglaan 1, 9713 AV Groningen, the Netherlands

## Abstract

**Background:**

Common mental disorders, such as depression, anxiety disorder, and adjustment disorder, have emerged as a major public and occupational health problem in many countries. These disorders can have severe consequences such as absenteeism and work disability. Different interventions have been developed to improve the return-to-work of employees with common mental disorders, but still a large proportion of employees experiences health and work problems after their return-to-work. For this reason, the SHARP-at work intervention is developed to prevent a relapse of sickness absence among employees who have returned to work after a period of sickness absence because of common mental disorders. We aim to evaluate the effectiveness, cost-benefit and process of the intervention compared to care as usual.

**Methods/Design:**

The study is designed as a cluster-randomised controlled trial with randomisation at the level of the occupational physician. Employees who have returned to work after a period of sickness absence because of a common mental disorder are included in the study. Employees in the intervention group will receive the SHARP-at work intervention. The intervention focusses on active guidance of employees by occupational physicians during the first weeks of work after sickness absence. Employees in the control group will receive care as usual. Outcomes will be assessed at baseline and at 3, 6, and 12 months follow-up. The primary outcome is cumulative recurrent sickness absence days. Secondary outcome measures are mental health, work functioning, and coping. Adherence to the protocol, communication between stakeholders, and satisfaction with the treatment are the process measures assessed in both study groups. Cost-benefit is calculated from a societal perspective. Finally, prognostic factors for a relapse of sickness absence are investigated.

**Discussion:**

This study goes beyond return-to-work by focussing on the prevention of recurrent sickness absence. The study incorporates not only outcomes on sickness absence and mental health but also on health-related work functioning. The results of this study can contribute to a further development of practice guidelines and the promotion of sustainable work participation.

**Trial registration:**

NTR1963

## Background

Common mental disorders (CMDs), such as depression, anxiety disorder, and adjustment disorder, have emerged as a major public and occupational health problem in many countries [1]. Several studies have found a relationship between CMDs and long term sickness absence and work disability [2-7]. In the Netherlands, about one in every three new work disability benefit recipients is disabled for work because of mental health problems [8,9]. The increase in sickness absence and work disability because of CMDs has serious negative economical consequences calling for preventive action [3,5,7,10,11].

Recently, attention is also given to the at-work decrements in performance because of CMDs, which seems to be even more costly than absenteeism [1,6,12-15]. In their review, Lerner and Henke show that depression is significantly associated with a reduction of job performance and productivity; it was demonstrated that employees with a significant improvement in depression still have more trouble with performing well compared to their healthy colleagues [6]. These findings emphasize the importance of interventions aiming at employees with CMDs who are at work. Yet, most interventions for employees with mental health problems are curative and focus on reintegration [16-20]. No interventions exist which aim at providing support after return-to-work although it is known that employees who return to work often are not fully recovered from their initial complaints [18-20]. Moreover, research in the Netherlands showed that one out of five employees who have returned to work after a sickness absence period because of a CMD experiences a relapse of sickness absence due to CMDs (Koopmans et al., submitted for publication). For these reasons, we have developed an intervention aiming at the prevention of a relapse of sickness absence among employees who have (partially/fully) returned to work after a period of sickness absence because of a CMD.

The intervention is called "SHARP-at work". SHARP is an acronym for Stimulating Healthy participation And Relapse Prevention. The intervention is based on the guideline "Management of mental health problems of workers by occupational physicians" of the Netherlands Society of Occupational Medicine. This evidence-based guideline, developed in 2000 and revised in 2007, is introduced to facilitate the return-to-work of employees on sickness absence because of mental health problems [21,22]. The goal of the guideline is to activate the employee when stagnation occurs in the process of problem identification, problem solving, and implementation of solutions regarding issues that caused sickness absence and factors that hinder return-to-work. By this, employees learn to control their own recovery. The guideline has shown to be effective in reducing the number of employees who are on long-term sickness absence because of CMDs [18,23,24].

The SHARP-at work intervention is developed to improve problem solving strategies regarding problems or opportunities at work for employees who have returned to work. Successful implementation of solutions is stimulated by guiding employees in involving their line manager (i.e. the supervisor). This intervention will be implemented by the occupational physicians (OPs) of the employees. The goal of the intervention is the prevention of a relapse of sickness absence and improving mental health and work functioning.

Given the serious consequences of CMDs for the individual employee and the high social and economic costs for the workplace, the employer, the health system, and society, the promotion of sustainable work participation among employees with CMDs is very important. Therefore, the primary aim of this study is to evaluate the effectiveness of the SHARP-at work intervention compared to care as usual (CAU) in preventing a relapse of sickness absence among employees who have returned to work after a period of sickness absence because of a CMD. We hypothesise that employees who return to work, after sickness absence because of a CMD, and undergo the SHARP-at work intervention will have less recurrent sickness absence days compared to employees who receive CAU. Secondary aims are to improve mental health and work functioning and to stimulate better coping mechanisms. In addition, the cost-benefit of the intervention will be examined. Along with these evaluations, we will conduct a process evaluation among employees, OPs, and line managers. Finally, prognostic factors for a relapse of sickness absence will be investigated. To our knowledge, this is the first study focussing on guiding employees at work after they have returned to work because of a period of sickness absence due to CMDs.

## Methods/Design

The CONSORT statement and the extension for cluster randomised trials is used to describe the design of the study [25,26].

### Study context

In the Netherlands, both the employer and the employee are responsible for return-to-work. According to the Dutch Gate Keeper Act, the employer has a two-year obligation to pay an employee on sickness absence. After this period, the employee can apply for work disability benefit. During the first two years of sickness absence, the employer and the employee have to make all efforts possible to realise a return-to-work for the employee. For this reason, the employer is obliged to contract an occupational physician (OP). The employee has to visit the OP when being on sickness absence. OPs treat employees according to guidelines. For CMDs, the evidence-based guideline of the Netherlands Society of Occupational Medicine is used to support employees on sickness absence because of mental health problems [21,22]. The OP and the employee should be meeting each other regularly as long as the employee has not fully returned to work. After full return-to-work, at least one meeting should take place to focus on relapse prevention. In practice, OPs do not seem to act upon this last step of the guideline (Rebergen et al., submitted for publication).

### Study design

**Figure 1:**
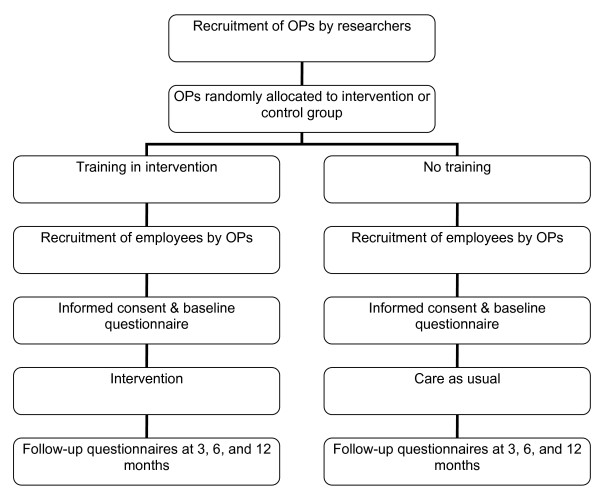
**Study design**.

The study is designed as a two-armed cluster-randomised controlled trial for the prevention of a relapse of sickness absence by the SHARP-at work intervention compared to CAU (Figure 1). The study is conducted among employees who have returned to work after a period of sickness absence because of a CMD. Randomisation occurs at the level of the OP because employees can not be randomly assigned to OPs and OPs can not be expected to provide both guidance according to the SHARP-at work intervention and CAU. OPs, who have given their consent for participation, are randomised in the intervention or control group. OPs in the intervention group receive training in the SHARP-at work intervention. OPs in the control condition do not receive this training until the end of the study period and provide CAU.

Employees will be included from December 2009 to December 2010. OPs will invite an employee to participate in the study if the employee is diagnosed with a CMD at the start of the sickness absence period and ready to return to work. Employees treated by an OP trained in the intervention will receive the intervention. Employees who are treated by an OP in the control group will receive CAU. Regardless of the treatment group, employees still have the possibility to be treated simultaneously by other health care workers.

The Medical Ethical Board of the University Medical Center Groningen has given approval for the study design, the research protocol, questionnaires, information letters, and the informed consent. Employees can participate voluntarily in this study. They are informed that they can leave the study at any time without consequences. All employees sign an informed consent. If an employee drops out, care will be continued.

### Recruitment of occupational physicians

Recruitment of OPs takes place in collaboration with ArboNed, one of the largest Occupational Health Services (OHS) in the Netherlands. OPs participating in this study are employed at this OHS and are affiliated with companies of different sizes, in different sectors and in different parts of the Netherlands. Company size ranges from less than five employees up to more than a 1000 employees in different sectors, for instance industry, education, health care, and customer services. All regions in the Netherlands, except the south, are participating in the study. OPs are excluded when they: 1. have an upcoming retirement, resignation, sabbatical, or pregnancy leave, or 2. are unable to use the internet and/or email.

### Recruitment of participants

#### Inclusion criteria

Employees participating in the study are between 18 and 63 years old and employed in a paid job. Furthermore, they have: 1. a diagnosis of a CMD given by their OP at the start of the sickness absence period, 2. a period of sickness absence due to a CMD of at least two weeks, and 3. a planned return-to-work within two weeks.

#### Exclusion criteria

Employees are excluded when: 1. the present sickness absence spell has been longer than 12 months, 2. they have had a period of sickness absence due to a CMD three months prior to the present sickness absence spell, 3. they have severe mental disorders, like psychotic disorders, bipolar disorder, or post-traumatic stress disorder, 4. they have somatic complaints or disorders that have a predominant influence on work disability, 5. they are pregnant or have an upcoming retirement, resignation, or layoff, and 6. they are unable to speak, read, write, or understand the Dutch language.

#### Procedure

Employees are recruited by the OPs participating in the study. The OP checks all inclusion and exclusion criteria. The diagnosis of a CMD has been made by the OP at the start of the sickness absence period. OPs are trained in diagnosing mental disorders and use a nationwide coding system, Classification of Diseases (in Dutch: CAS) [27], based on the International Classification of Diseases [28].

If the employee is interested to participate in the study, the OP asks whether contact information of the employee can be given to the researcher (IA) and hands over an information folder about the study. After the employee has received the information and has given approval for the researcher to make contact, the OP gives the contact information to the researcher. The researcher contacts the employee and asks if the employee would like to participate in the study. If the employee is willing to participate, informed consent and the baseline questionnaire (electronic or paper version) are send to the employee with a postage paid envelope.

When the informed consent and the baseline questionnaire are filled in and returned to the researcher, OPs in the intervention group are informed that the intervention can be started. OPs in the control group keep on treating their employees according to CAU. Follow-up questionnaires are send to the employee at 3, 6, and 12 months. At these points in time, administrative data on cumulative sickness absence days are also collected by means of the registry system of the OHS.

### Intervention

#### Training of occupational physicians in the intervention group

OPs receive a two-day training in the SHARP-at work intervention. Training is provided by experienced trainers in occupational health interventions.

#### Treatment of participants in the intervention group

The intervention consists of five steps the employee has to undertake when return-to-work is started. The OP monitors that the employee follows these steps and uses interventions to activate the employee when needed. The five steps are delineated below.

1. Make an inventory of problems and/or opportunities encountered at work

2. Brainstorm on solutions

3. Write down the solutions and the support needed and assess the applicability

4. Discuss the solutions with the line manager and make an action plan

5. Evaluate the action plan and the implementation of solutions

The OP can use assignments for the employee to write down and structure the process. In general, the OP will counsel on the process level. (S)he does not discuss the content of problems and solutions but challenges the employee to reflect on the relative seriousness of problems and the feasibility of solutions. This can be done by asking questions that stimulate the employee to think about possible perspectives. This form of guidance is related to Socratic questioning. The intervention differs from the guideline of the Netherlands Society of Occupational Medicine by the emphasis on problems and possibilities encountered *at work *when an employee has already returned to work.

### Care as usual

#### Training of occupational physicians in the control group

All OPs participating in the study are trained in the guideline of the Netherlands Society of Occupational Medicine. There is no additional training of OPs in the control group as part of this study. At the end of the study period these OPs will be trained in the SHARP-at work intervention if it proves to be effective.

#### Treatment of participants in the control group

CAU is delivered according to the evidence-based guideline of the Netherlands Society of Occupational Medicine [21,22]. CAU comprises guidance in regaining control and activating problem solving when the employee is still on sickness absence and at least one consultation session after return-to-work, addressing relapse prevention.

### Sample size

For the power calculation, recurrent sickness absence days are considered the primary outcome measure. To calculate the sample size, administrative data from the sickness absence registry of the OHS was used. From 2001 to 2007, recurrent sickness absence days among employees, who had returned to work after a period of sickness absence because of CMDs, were registered. For the power calculation, only recurrent sickness absence days of the first recurrence episode until 1 year later were used to approximate the 1 year follow-up of the present study (N = 4443). The variance in recurrent sickness absence days at the level of the OP was taken into account.

During the above-mentioned period, the mean days of recurrent sickness absence was 68.5 (standard deviation is 119.6). The target of the present study is to reduce the recurrent sickness absence days with 20%, i.e. an average of 12.7 recurrent sickness absence days per employee per year. The OPs in this OHS's dataset were randomly divided into two groups. One group was called "the intervention group" from which 20% of the recurrent sickness absence days was subtracted to create the difference between the intervention and control group and to calculate an effect size.

For a decrease of 12.7 recurrent sickness absence days per employee during 1 year at alpha = 0.05 and ICC = 0.05, 50 OPs, each providing five employees, need to be included in each group (the intervention and control group) [29]. The five employees must be viewed as the average number per OP. For this multilevel power calculation, an effect size of 0.18 was taken into account. This effect size was calculated by using a logtransformation on the data to create a normal distribution and subsequently by dividing the difference in mean days of recurrent sickness absence between the intervention and control group (difference is 0.25) by the standard deviation (standard deviation of difference is 1.4). To include the effect size in the multilevel power calculation, it had to be transformed to a correlation coefficient, which resulted in a correlation of 0.09 [30].

The collaborating OHS serves approximately one million insured employees. A total of 350 OPs is working for this OHS and each OP serves around 2500 to 3000 employees. Of these employees it is estimated that 1.3% (30% of the national sickness absence rate among Dutch employees of 4.3%) will be on sickness absence because of a CMD during a 1 year period [31]. Therefore, an OP will see around 32 to 39 employees per year who are absent because of CMDs. Following this, the source population of the OHS is large enough to recruit the required number of OPs and employees according to the sample size calculation.

### Randomisation and treatment allocation

Employees can not be randomly assigned to OPs trained in the intervention or OPs not trained in the intervention because the OPs and employees are bound to each other by the company. It is also impossible to train all OPs and to let them randomly apply the intervention or CAU to employees because of the risk of contamination. Therefore, randomisation occurs at the level of the OP. OPs who have given their consent to participate in the study are randomly assigned to the intervention group or the control group. To ensure a good contrast between these two groups, OPs in the intervention group are specifically asked not to talk about the intervention with OPs in the control group. Additionally, the OPs in the intervention group have two feedback meetings to discuss the application of the intervention with each other.

A computerised random allocation sequence for randomising the OPs is developed by an independent researcher. When all OPs are recruited, the independent researcher, who is blind to the identity of the OPs, uses the allocation sequence to randomise the OPs. After this, the allocation of the OPs can not be changed and the independent researcher informs the researchers about the allocation of the OPs. The allocation of employees follows from the allocation of their OP. Employees with an OP in the intervention group are automatically allocated to the intervention group and employees with an OP in the control group are automatically allocated to the control group.

### Blinding

Validity can be threatened if employees in the intervention and control group would know about the other group. Because this study is a pre-randomised trial, in which the employees are already randomised before informed consent is given, different information about the study can be provided to the intervention and control group [32]. To ensure that employees are not aware and stay unaware of the two study conditions, the OPs are requested not to talk about this with the employees. Whereas employees are blinded for treatment allocation, blinding of allocation for OPs is not possible because they will know if they are trained in the intervention or not.

### Primary outcome

The primary outcome measure is a relapse of sickness absence, measured as cumulative recurrent sickness absence days. A relapse is defined as a 30% decrease in working days per week or a decrease of at least one day per week because of sickness absence. Recurrent sickness absence days are operationalised as days of sickness absence among employees who have worked a steady amount of days during the first two weeks after they have returned to work. This information will be obtained by record linkages with the sickness absence registry of the OHS and by the employee questionnaires.

### Secondary outcomes

#### Mental health problems

The Four-Dimensional Symptom Questionnaire (4DSQ) is used to measure symptoms of distress, depression, anxiety, and somatisation. The 4DSQ is a self-report questionnaire of 50 items and measures distress, depression, anxiety, and somatisation. The 4DSQ has been validated in a primary care and working population [33,34].

The HADS is a 14-item self-report questionnaire used to measure depression (7 items) and anxiety (7 items) [35]. The questionnaire can be used in somatic, psychiatric, and primary care patients, as well as in the general population [36] and in working populations [37]. The HADS has been validated in different groups of Dutch subjects [38].

#### Work functioning

Work functioning is measured by the Work Role Functioning Questionnaire (WRFQ) [39,40]. The questionnaire has been cross-culturally adapted and translated into Dutch and pre-tested in a working population (Abma et al., submitted for publication). Results of the pre-test showed that the cross-cultural adaptation was successful. The WRFQ measures the perceived difficulties in meeting work demands among employees given their physical health or emotional problems. The WRFQ consists of 27 items divided into five subdomains: 1. work scheduling demands, 2. output demands, 3. physical demands, 4. mental demands, and 5. social demands.

#### Coping behaviour

Coping behaviour is measured by the 19-item version of the Utrecht Coping List (UCL) which assesses coping styles [41]. The questionnaire consists of the following five (coping style) scales: 1. active problem-focussing, 2. seeking social support, 3. palliative reaction pattern, 4. avoidance behaviour, and 5. expression of emotions.

### Economic evaluation measures

Along with the sickness absence data from the OHS's registry system, administrative data of the OHS on consultations of the employee with the OP and company welfare workers is collected. Furthermore, the Trimbos/iMTA questionnaire for Costs associated with Psychiatric Illness (Tic-P), a validated Dutch questionnaire, is used to measure medical consumption and at-work decrements in performance [42]. Finally, an extra item on out-of-pocket costs is added to the Tic-P to calculate medical expenses that are not covered by health insurance.

### Process evaluation measures

A process evaluation is conducted to examine a) the appraisals, attitudes, and activities of OPs, employees, and line managers in the intervention and control group during the treatment period and b) whether OPs adhere to their protocol. OPs who are trained in the intervention, receive a questionnaire before and after the training to examine the quality of the training and the skills and attitudes of the OPs. For the intervention group, a questionnaire is developed for the OP and the employee at baseline. These questionnaires contain items on readiness for change concerning the intervention. At 3-months follow-up, the employees and the OPs in both the intervention and control group receive a questionnaire about the process of treatment during the first three months of return-to-work. The questions elaborate on what was discussed during the consultations, whether assignments were given, which assignments were made, satisfaction with the treatment, and communication between the employee, OP, and line manager. For the intervention group, a questionnaire is also developed for the line manager. The line manager receives a questionnaire on readiness for change before a meeting takes place with the employee as part of the intervention. At 3-months follow-up, the line manager receives a second questionnaire with the same questions as the questionnaires at 3 months for the employee and the OP.

### Prognostic measures

Research on prognostic variables for a relapse of sickness absence has only been conducted in a few studies. For this reason, a range of variables is included in this study to investigate prognostic factors for a relapse of sickness absence. At baseline, the following variables are measured: personal characteristics (e.g. age, gender, marital status, educational level, and physical health), job characteristics (e.g. tenure, size of the company, sector, profession, contract type, number of contract hours before and after sickness absence, and work accommodations after return-to-work), and psychosocial work characteristics (job demands, decision latitude, social support [43,44], job insecurity, conflicts, emotional load [45], work engagement [46], and expectancy to stay-at-work).

### Statistical analyses

Due to the multilevel design of the study (i.e. employees are nested in OPs), multilevel regression analyses will be performed according to the intention-to-treat principle. Per protocol analyses will be conducted to explore if deviations from the protocol have caused bias. Descriptive statistics will be used to measure differences in baseline characteristics between the intervention and control group. In case of significant differences, these will be controlled for in the effect evaluations.

#### Effect evaluation

The primary outcome variable "cumulative recurrent sickness absence days during follow-up" will be compared between the intervention and control group in the multilevel regression analyses. Time until a relapse of sickness absence will be examined by using Cox proportional hazard analysis to estimate hazard ratio's for a relapse and the 95% confidence interval. In case no software is available to conduct these analyses in a multilevel structure, cluster level survival analyses will be conducted with means for each cluster. In these analyses, cluster size will be introduced as a weighting factor. To investigate differences between the intervention and control group in changes (improvement) on all secondary outcomes, multilevel longitudinal analysis will be used. Pre-planned subgroup analyses on type of CMD, line manager participation in the study, size of the company, perceived decision latitude, type of work, and expectancy to stay-at-work will be conducted.

#### Economic evaluation

The economic evaluation will be performed as a cost-benefit analysis from a societal perspective. For this evaluation, the primary outcome measure, i.e. the number of recurrent sickness absence days after return-to-work, will be expressed in monetary terms. The time window will be from return-to-work until 1 year follow-up. Discounting will not be applied.

Both direct and indirect costs will be measured and valued. Costs of the intervention will be calculated by using the hourly wages of OPs. All contacts between the OP and the employee will be registered. The costs of health care consumption outside the intervention will be calculated by using tariffs of Dutch Guideline prices [47] based on information collected by the Tic-P questionnaire [42]. Out-of-pocket costs made by the employees in relation to their condition will also be included. The indirect costs of production losses due to sickness absence and presenteeism will be calculated by using the Friction costs method [48,49] according to the Dutch guidelines for economic evaluation [47].

## Discussion

This study is designed to investigate if employees, who have returned to work after a period of sickness absence because of a CMD, benefit from extra support during their first weeks of return-to-work. Effectiveness analyses will be performed to examine if the SHARP-at work intervention is successful in reducing recurrent sickness absence days compared to CAU. A cost-benefit analysis will be conducted to evaluate if the intervention is efficient, e.g. if better results are not at the expense of higher costs. Furthermore, the process of the intervention will be evaluated by inquiring employees, OPs, and line managers on adherence to the protocol and satisfaction with the treatment. Finally, prognostic factors for a relapse of sickness absence will be investigated.

### Methodological considerations

This study design has important strengths. First of all, the cluster-randomisation diminishes the risk of contamination of employees participating in the study. OPs are randomised in the intervention or control group, i.e. they will only treat employees, participating in the study, according to the SHARP-at work intervention or CAU. Furthermore, OPs in the intervention and control group work for different companies or for different departments of big companies. Hence, it is unlikely that employees in the intervention group will get in contact with employees in the control group. Additionally, the pre-randomisation in the cluster design makes it possible to blind employees for the study condition. Another strength of this study is the combination of objective and subjective data. Recurrent sickness absence days are not only measured by asking the employees on days of sickness absence after their return-to-work, but also by the OHS's sickness absence registry system. Finally, the study covers a large geographical area in the Netherlands and companies of different sizes and sectors are included. This will make it possible to generalise the results to a relatively large working population.

A limitation of the study is the inclusion of participants by the OPs. The possibility exists that OPs in both study groups select those employees who have, in their view, good potential for fast recovery and small chances for a relapse of sickness absence. Although it has been stressed to all OPs to invite all employees eligible for the study, we can not exclude the possibility of bias. Questions to investigate if participating employees were thought to be more suitable for the study are included in the process evaluation for OPs. Another possibility for bias to occur is the selection of employees who have a good relationship with their OP. Unfortunately, we can not exclude this bias, but we ask questions in the process evaluation on the relationship between the OP and the employee. Finally, recruiting 500 employees for the study is a challenge. Most studies in this field have problems with recruiting participants. We have tried to minimise these problems by embedding this study in one large OHS. All participating OPs work for the same OHS and are stimulated to contribute to the study by this OHS. Moreover, we expect fewer refusals to participate because OPs invite employees to participate in the study. The OP has already built a relationship with the employee which will contribute to a safe environment to make the choice to participate in the study.

### Relevance/impact of results

This study goes beyond return-to-work by focussing on sustainable work participation after return-to-work. It incorporates not only outcomes on sickness absence and mental health but also on health-related work functioning. The study has a high societal relevance because costs for sickness absence could be lowered and a sustainable working life could be facilitated. Furthermore, OPs and other occupational health professionals may benefit from the intervention as it could serve as an extension of the already existing guideline of the Netherlands Society of Occupational Medicine. Results of the study will become available in 2012.

## Abbreviations

CMD(s): common mental disorder(s); SHARP: Stimulating Healthy participation and Relapse Prevention; CAU: care as usual; OP(s): occupational physician(s); OHS: occupational health service; 4DSQ: Four-Dimensional Symptom Questionnaire; HADS: Hospital Anxiety and Depression Scale; WRFQ: Work Role Functioning Questionnaire; UCL: Utrecht Coping List; Tic-P: Trimbos/iMTA questionnaire for Costs associated with Psychiatric Illness.

## Competing interests

The authors declare that they have no competing interests.

## Authors' contributions

IA, JvdK, and UB designed the study. IA and JvdK developed the intervention. IA is responsible for the data collection and drafted the manuscript. All authors provided comments on the drafts of the manuscript and approved the final version of the manuscript.

## Pre-publication history

The pre-publication history for this paper can be accessed here:

http://www.biomedcentral.com/1471-2458/10/132/prepub
